# Storage Survival and Thermal Inactivation of *Listeria monocytogenes* in Peanut Butter and Whole Egg Powders

**DOI:** 10.3390/foods15183174

**Published:** 2026-09-08

**Authors:** Laura Muñoz, Luis Sabillón

**Affiliations:** 1 Department of Family & Consumer Sciences, New Mexico State University, Las Cruces, NM 88003, USA; 2 Center of Excellence in Sustainable Food and Agricultural Systems, New Mexico State University, Las Cruces, NM 88003, USA

**Keywords:** low-moisture foods, desiccation survival, isothermal inactivation, Weibull model

## Abstract

*Listeria monocytogenes* is a concern in low-moisture foods because it can survive prolonged dry storage conditions and exhibit increased heat resistance at low water activity (a_w_). This study evaluated the survival and thermal resistance of *L. monocytogenes* in peanut butter powder and whole egg powder equilibrated to approximately 0.25 and 0.45 a_w_. Inoculated powders were stored at 22 °C for 16 months, followed by thermal treatment at 75 and 80 °C. Log-linear, biphasic, and Weibull models were compared using goodness-of-fit metrics and information criteria. Weibull scale parameters (*δ*) were calculated from replicate-level fits. During storage, *L. monocytogenes* persisted longer at 0.25 a_w_ than at 0.45 a_w_ in both matrices. In peanut butter powder, populations declined from 8.48 to 2.70 log CFU/g at 0.45 a_w_ and from 8.89 to 3.64 log CFU/g at 0.25 a_w_ after 16 months. In whole egg powder, populations decreased from 8.25 log CFU/g to nondetectable levels by month 15 at 0.45 a_w_, whereas 4.62 log CFU/g remained after 16 months at 0.25 a_w_. Nonlinear models generally outperformed the log-linear model. The largest modeled time to the first 1-log reduction (*δ*) occurred at 75 °C and 0.25 a_w_ in whole egg powder (18.28 min; 95% CI: 16.34–20.45) and peanut butter powder (13.38 min; 95% CI: 9.18–19.52). Lower a_w_ enhanced both long-term survival and thermal resistance of *L. monocytogenes*, demonstrating the importance of a_w_- and matrix-specific validation of thermal processes for low-moisture foods.

## 1. Introduction

Low-moisture foods (LMFs) have historically been viewed as low risk because they do not support pathogen growth, yet outbreaks and recalls have shown that these products can harbor pathogens for long periods and can remain important vehicles for foodborne illness [1]. While *Salmonella* has traditionally been the primary pathogen of concern in LMFs, increasing recalls involving *L. monocytogenes* in products such as nuts, seeds, and flours have heightened industry and regulatory attention [2,3,4]. Current evidence indicates that *L. monocytogenes* can survive for extended periods in dry environments and may become highly thermotolerant after adaptation to low water activity (a_w_) [4,5,6,7].

The mechanistic basis for bacterial survival in LMFs is multifactorial. Reduced water availability limits molecular mobility and slows heat-induced damage to essential cellular structures, while osmotic and desiccation stress activate broad adaptive responses that improve survival under subsequent lethal conditions [5,8]. In *L. monocytogenes*, osmotic adaptation depends heavily on the uptake of compatible solutes [3], particularly glycine betaine and carnitine, and the expression of these uptake systems is linked to the σ^B-mediated general stress response [9,10,11]. These adaptive systems help explain why prior exposure to low-a_w_ environments can increase the robustness of *L. monocytogenes* in foods and food-processing settings.

Empirical studies in dry food systems support these concepts. In peanut-containing products, *L. monocytogenes* was shown to survive for at least 24 weeks and to display greater thermal resistance in low-a_w_, high-fat matrices such as peanut butter and chocolate-peanut spread than in higher-moisture liquid products [12]. Likewise, in almond meal, *L. monocytogenes* remained stable during long-term storage and exhibited markedly greater heat resistance at 0.25 a_w_ than at 0.45 a_w_ [13], demonstrating the strong protective effect of low a_w_ in a nut-based low-moisture ingredient. Similar a_w_-dependent behavior has also been reported in non-fat dry milk powder, wheat flour, and cocoa powder, where survival during storage and thermal resistance during heating both increased as a_w_ decreased [14,15,16]. An inverse relationship between a_w_ and thermal resistance is typically observed; however, this relationship is strongly influenced by food matrix composition (e.g., fat and protein content) [17]. Collectively, these findings indicate that low moisture does not simply prevent growth; it can also create a state in which *L. monocytogenes* remains viable and more difficult to inactivate.

Despite growing interest in *L. monocytogenes* in LMFs, the available data remain much more limited than those for *Salmonella*, and further work is needed in representative dry ingredients to support science-based lethality validation. Peanut butter powder and whole egg powder are particularly relevant matrices for this purpose because they are low-moisture ingredients with distinctly different proximate compositions, providing an opportunity to examine how a_w_ and matrix composition jointly affect *L. monocytogenes* survival and thermal resistance. Accordingly, the objectives of this study were to (i) evaluate the long-term survival of *L. monocytogenes* in peanut butter powder and whole egg powder equilibrated to two target a_w_ levels during storage at 22 °C, (ii) characterize its thermal inactivation kinetics in these matrices during isothermal treatments (75–80 °C), (iii) compare the performance of alternative primary inactivation models for describing the observed survivor curves, and (iv) determine the influence of a_w_, temperature, and matrix composition on D-values for process interpretation and validation. By integrating storage survival and thermal resistance data across compositionally distinct matrices, this study provides critical insights to support the development and validation of control strategies for *L. monocytogenes* in LMF systems.

## 2. Materials and Methods

### 2.1. Low-Moisture Food Matrices and Proximate Analyses

Two commercially available low-moisture food (LMF) powders, peanut butter and whole egg, were obtained from Augason Farms (Salt Lake City, UT, USA) in approximately 2-pound aluminum cans and stored at 22 °C until use. Proximate composition analyses (i.e., moisture, crude protein, crude fat, crude fiber, carbohydrates, and ash) were conducted by New Jersey Feed Laboratory (Ewing, NJ, USA) using standard AOAC International methodologies for feed and food analysis. Moisture was determined by oven drying (AOAC 934.01), crude protein by combustion (AOAC 990.03), crude fat by ether extraction (AOAC 920.39), crude fiber by AOAC 978.10, and ash by dry incineration (AOAC 942.05). Carbohydrate content was calculated by difference. The results of the proximate composition analyses are presented in Table 1. The whole egg powder contained < 2% sodium silicoaluminate as an anti-caking agent, while the peanut butter powder contained sugar and non-iodized salt.

The proximate-composition values in Table 1 were obtained from subsamples from the same commercial lots used in the microbiological experiments. Accordingly, the values directly characterized the experimental lots.

### 2.2. Bacterial Strains

A five-serotype cocktail of *Listeria monocytogenes* was used, consisting of strains 4b (ATCC 19115), 1/2a (ATCC 19111), 3a (ATCC 19113), 1/2b (ATCC BAA-839), and 1/2c (ATCC 51779), obtained from the American Type Culture Collection (ATCC; Manassas, VA, USA). Stock cultures were maintained at −80 °C in Brain Heart Infusion (BHI; Research Products International, Mt. Prospect, IL, USA) supplemented with 20% (*v*/*v*) glycerol. Prior to use, each strain was twice propagated in BHI broth at 35 ± 1 °C for 24 h. Cultures were streaked onto Tryptic Soy Agar supplemented with 0.6% yeast extract (TSAYE; Hardy Diagnostics, Santa Maria, CA, USA) and incubated at 35 ± 1 °C for 24–48 h to obtain isolated colonies. Working cultures were stored at 4 °C and used within 30 days to prepare the inoculum for subsequent experiments.

### 2.3. Inoculum Preparation

Individual colonies from working stock plates were inoculated into 9 mL BHI broth and incubated at 35 ± 1 °C for 24 h. Subsequently, 300 µL aliquots were spread onto TSAYE plates and incubated for 24 h to obtain confluent lawns. Cells were harvested by adding 5 mL of 0.1% buffered peptone water (BPW; Difco™, Sparks, MD, USA) and gently scraping the surface with a sterile L-shaped cell spreader. Equal volumes of each strain suspension were combined in a sterile conical tube and vortexed (30 s) to form a five-strain cocktail. The mixture was centrifuged (5000× *g*, 10 min, 4 °C), washed three times with BPW, and resuspended in 4 mL BPW to obtain an approximate concentration of 10^11^ CFU/mL for subsequent experiments.

### 2.4. Long-Term Survivability and Thermal Inactivation Study

#### 2.4.1. Sample Inoculation and Water Activity Equilibration

Samples (300 g) were inoculated using a seed inoculation method adapted from Minor et al. [18]. Briefly, 30 g of product was inoculated with 3 mL of the *L. monocytogenes* cocktail and manually mixed for 10 min in a sterile 3-mil polyethylene blender bag. The inoculated portion was then blended with 270 g of uninoculated product to achieve a target level of 10^8^–10^9^ CFU/g. Samples were evenly spread onto sterile aluminum trays (14 × 10 in) and equilibrated in a controlled environmental chamber (BINDER Inc., Bohemia, NY, USA) at target a_w_ values of 0.25 and 0.45 for 7 days at 22 ± 1 °C. Water activity was monitored daily using a calibrated meter (LabSwift-a_w_, Novasina AG, Lachen, Switzerland), and samples were used for thermal inactivation and long-term survivability studies once the target a_w_ (±0.02) values were reached. All reported a_w_ values represent measurements performed at approximately 22 °C and therefore reflect the water activity of the samples under storage and equilibration conditions, not the temperature during thermal treatment. Two independently inoculated 300 g batches were prepared for each matrix and a_w_ condition and served as biological replicates. Inoculum uniformity was verified by analyzing triplicate 2 g samples collected immediately after inoculation and during equilibration at days 1, 4, and 7. The *Listeria* populations in these samples were quantified through serial dilution and enumeration on TSAYE plates.

#### 2.4.2. Storage of Inoculated Products

Following equilibration to the target a_w_, inoculated samples were portioned into 10 g aliquots and placed in moisture-barrier foil locking bags (PELCO^®^, Ted Pella, Inc., Redding, CA, USA). Samples were stored in an incubator at 22 ± 1 °C for up to 16 months. Microbial survival was assessed every other day during the first week, weekly throughout the first month, biweekly during the second month, and monthly thereafter. At each time point, triplicate samples were analyzed for *Listeria* counts and a_w_, with a_w_ measurements performed at approximately 22 °C.

#### 2.4.3. Isothermal Heat Treatments

After equilibration, 0.40 ± 0.02 g samples were loaded into aluminum thermal death time (TDT; model TDT3) cells acquired from Washington State University [19]. Samples were subjected to isothermal treatments at 75 °C or 80 °C in a circulating water bath (Thermo Scientific™, Waltham, MA, USA). The temperature of the water bath was monitored independently with a calibrated K-type thermocouple thermometer (H-B DURAC, SP Bel-Art™, Norwalk, CT, USA). Come-up time (CUT), defined as the time required for the sample temperature to reach within 0.5 °C of the target temperature, was determined using T-type thermocouples positioned at the geometric center of TDT cells. Time–temperature history was recorded using a data logger (OM-CP-OCTPRO, Omega Engineering Inc., Norwalk, CT, USA). The CUT was 1.5 ± 0.2 min, after which the designated isothermal treatment time was initiated. Microbial concentrations were measured at predetermined sampling times, including the end of the CUT, and the population measured at the end of the CUT was used as the initial concentration for modeling the subsequent isothermal inactivation curves. Thus, any lethality occurring during the CUT was reflected in the measured bacterial concentration at the start of the modeled inactivation period rather than treated as a separate nominal treatment time.

At each sampling time, three analytical subsamples were taken from each biological replicate and served as technical replicates. Technical replicates characterized within-batch sampling and enumeration variability and were not treated as independent experimental units in inferential analyses. These samples were collected at five predetermined time points and immediately cooled in an ice-water bath for 1 min to stop thermal inactivation.

### 2.5. Enumeration of Listeria monocytogenes

#### 2.5.1. Spread Plate Method

Heat-treated samples were aseptically transferred to sterile 10 mL centrifuge tubes (Globe Scientific, Mahwah, NJ, USA) containing 3.6 mL of sterile 0.1% PBW to achieve a 10-fold dilution. The mixture was homogenized using a vortex for 1 min and further serially diluted to appropriate levels. Aliquots of 0.1 mL were spread onto TSAYE plates and incubated at 35 ± 1 °C for 3 h to facilitate the recovery of injured cells. Subsequently, plates were overlaid with approximately 10 mL of *Listeria* Selective Agar Base (Oxford [CM0856B]; Thermo Scientific™) enriched with a selective supplement (Oxoid™ [SR0206E]; Thermo Scientific™) to minimize the growth of unwanted microorganisms. Plates were incubated for 48 h at 35 ± 1 °C prior to enumeration. The same approach was used to enumerate the *Listeria* population during the long-term storage study.

#### 2.5.2. Viability PCR

Samples with *Listeria* plate counts below the detection limit (<10 CFU/g) were further evaluated using viability PCR (vPCR; GENE-UP, bioMérieux, Inc., Salt Lake City, UT, USA) to assess the presence of membrane-intact *L. monocytogenes* cells not recovered by direct plating. Samples were enriched in LPT broth at 35 ± 1 °C for 24 h and treated with BACTIVIA PMAxx™ (bioMérieux, Inc.) before qPCR analysis to reduce amplification of DNA from cells with compromised membranes. Procedures followed AOAC Official Method 2019.11 (GENE-UP^®^ LMO2) and the manufacturer’s BACTIVIA PMAxx protocol. Positive vPCR results were further evaluated by streaking enriched samples onto selective *Listeria* Oxford agar. Thus, direct plating assessed culturability in the original samples, whereas post-enrichment vPCR assessed *L. monocytogenes* associated with membrane-intact cells, and selective culture assessed recovery following enrichment.

### 2.6. Modeling Thermal Inactivation Kinetics

Thermal inactivation curves were generated by plotting bacterial survival as a function of treatment time. To characterize the inactivation behavior, the experimental data were fitted independently to three primary models: the log-linear, Weibull, and biphasic models using GInaFiT Microsoft Excel Add-ins Package (Version 1.8; [20]). GInaFiT supports both classical log-linear and non-log-linear survival models, thereby allowing comparison of linear and non-linear survivor curve behavior.

The log-linear model was expressed as

(1) $$Log\left(N_{t}\right)=Log\left(N_{0}\right)-k_{max}\cdot t$$

where *N_t_* is the bacterial population (log CFU/g) at time *t* (min), *N_0_* is the initial population (log CFU/g), and *k_max_* is the maximum specific inactivation rate (1/min). This model assumes that all cells within the microbial population have uniform sensitivity to the applied stress. In practice, however, this assumption is often violated due to inherent cell-to-cell heterogeneity. As a result, deviations from log-linear behavior are frequently observed, necessitating the use of nonlinear models for more accurate characterization of microbial inactivation.

The Weibull model was also fitted to the inactivation curves. The Weibull approach is widely used to describe concave, convex, and linear inactivation patterns and is often more suitable than the classical first-order model when non-linear survival behavior is observed [21]. The Weibull model was defined as

(2) Log(Nt)=LogN0−tδβ

where *N_t_* is the microbial population (CFU/g) at time *t*, and *N_0_* is the initial population. The *δ* represents the Weibull scale parameter (min), which influences the overall steepness of the survival curve. The parameter *β* is dimensionless and describes the curvature of the inactivation profile, with *β* < 1 indicating downward concavity (tailing), *β* > 1 indicating upward concavity (shouldering), and *β* = 1 corresponding to log-linear inactivation [22]. Under this parameterization, *δ* corresponds to the treatment time (min) at which the fitted curve reaches a 1 log reduction, because log (N_t_/N_0_) = −1 when *t* = *δ*. However, *δ* is not a classical constant D-value unless *β* = 1. For *β* ≠ 1, the time required for an x-log reduction is *t*_x_ = *δ*_x_^(1/*β*), and successive decimal reductions therefore do not occur at equal time intervals [21,22]. The Weibull model assumes that resistance to the applied treatment follows a Weibull distribution within the microbial population.

To account for inactivation curves showing an initial rapid decline followed by a slower reduction phase, the biphasic model was additionally evaluated. This model assumes that the population consists of two subpopulations with different levels of heat resistance: a major, more heat-sensitive fraction and a minor, more heat-resistant fraction. Such a model is particularly appropriate for survivor curves exhibiting biphasic or tailing behavior. The biphasic model was expressed as

(3) $$Log\left(N_{t}\right)=Log\left(N_{0}\right)+Log\;\left[f\cdot exp(-k_{max1}\cdot t)+\left(1-f\right)\cdot \left(-k_{max2}\cdot t\right)\right]$$

where *f* is the fraction of the initial population corresponding to the more heat-sensitive subpopulation, (*1* − *f*) is the fraction corresponding to the more heat-resistant subpopulation, and *k_max1_* and *k_max2_* are the specific inactivation rates (1/min) of the sensitive and resistant fractions, respectively. The biphasic model is useful for representing survival curves with two distinct inactivation phases and for capturing slope changes that cannot be adequately described by a single-rate model.

### 2.7. Model Discrimination and Statistical Analysis

Model discrimination was performed using a combination of statistical and mechanistic criteria to account for parameter complexity and goodness-of-fit, including root mean square error (RMSE), coefficient of determination (R^2^), adjusted R^2^_Adj_, Bayesian information criterion (BIC), and the corrected Akaike information criterion (AICc). For each condition, the model with the lowest AICc was considered the preferred model, whereas models differing by ΔAICc < 2 were considered to have comparable support. In addition to statistical performance, biological plausibility and parameter interpretability were also considered.

To support process-establishment analyses, D-values were calculated using the model that best characterized the inactivation behavior; which was the Weibull model. The log-transformed Weibull scale parameter (*δ*) were analyzed using a three-way analysis of variance (ANOVA) with matrix, a_w_, and temperature as fixed effects, including all two-way and three-way interactions. In addition, to facilitate interpretation within a_w_ levels, the data were stratified by a_w_ and analyzed separately using two-way ANOVA models with product and temperature as fixed effects. Each independently inoculated biological replicate was treated as the experimental unit. Technical replicate measurements were averaged within each biological replicate and were not treated as independent observations in the ANOVA. A separate Weibull curve was fitted to each independent biological replicate, yielding two replicate scale-parameter estimates per treatment. When significant main effects or interactions were detected, pairwise mean comparisons were conducted using Tukey’s honestly significant difference test at a significance level of *p* < 0.05.

## 3. Results

### 3.1. Proximate Composition and Initial Matrix Characteristics

The two low-moisture matrices differed markedly in composition prior to inoculation and storage (Table 1). Peanut butter powder had lower initial a_w_ and moisture content than whole egg powder (a_w_ 0.14 ± 0.01 vs. 0.21 ± 0.01; moisture 2.75 ± 0.19% vs. 3.95 ± 0.15%), and it was characterized by lower fat content (9.74 ± 0.10% vs. 36.86 ± 0.17%) and higher carbohydrate content (41.37 ± 0.14% vs. 9.51 ± 0.18%). Whole egg powder, in contrast, contained slightly more crude protein (44.95 ± 0.04% vs. 39.83 ± 0.02%) and substantially more fat than peanut butter powder.

### 3.2. Long-Term Survival of Listeria monocytogenes During Storage at 22 °C

**Figure 1:**
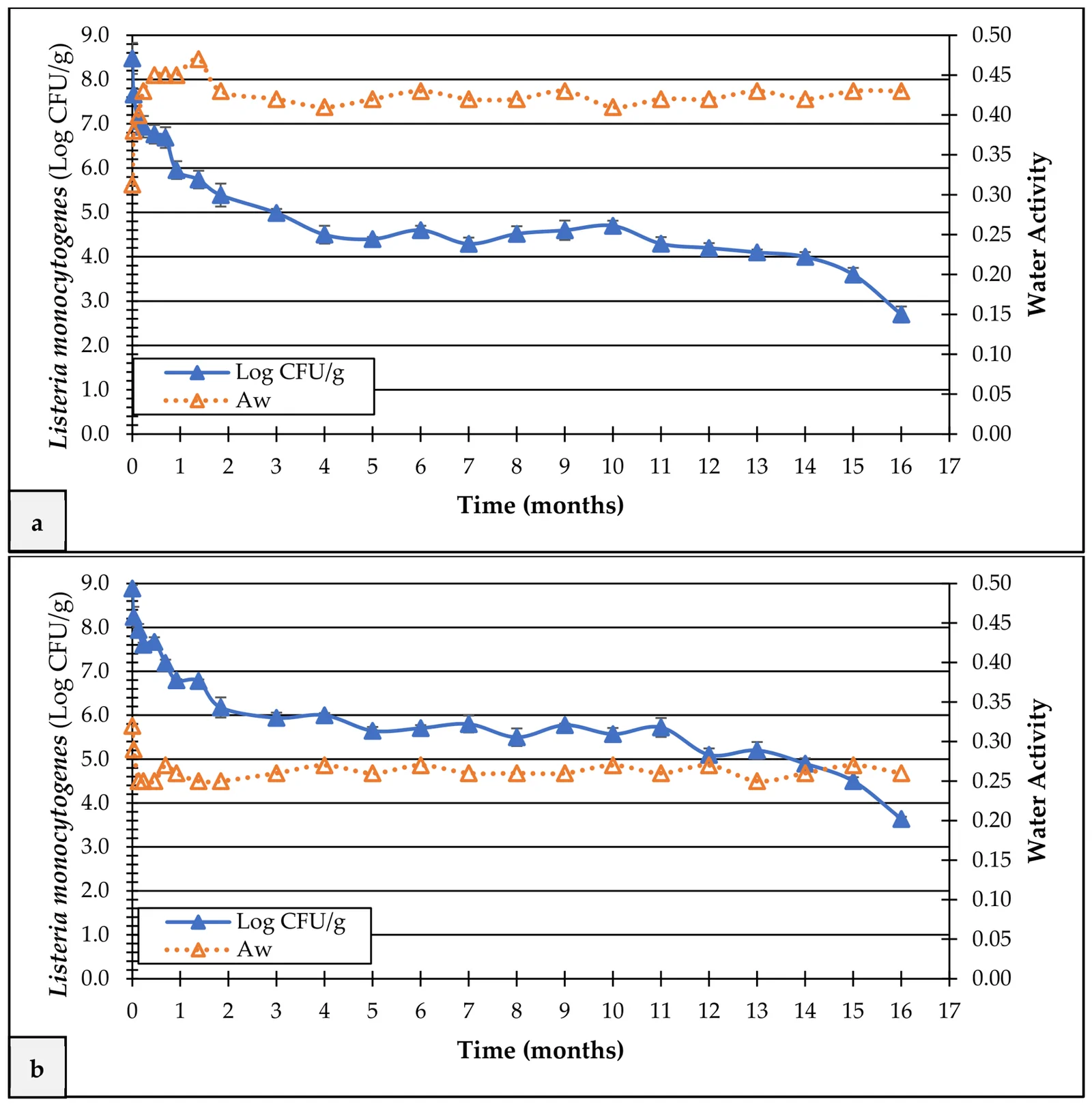
Survival of *Listeria monocytogenes* in peanut butter powder at different water activity (a_w_) levels during 16 months of storage at 22 °C. (**a**) 0.42 ± 0.03 a_w_; (**b**) 0.26 ± 0.02 a_w_. The solid (dark blue) line shows the population of *L. monocytogenes*, while the dashed (orange) line represents the a_w_ of peanut butter powder over storage time. Points represent mean log CFU/g values, and error bars represent the standard deviation of six replicates (n = 6; two biological replicates × three technical replicates per time point).

In peanut butter powder, *L. monocytogenes* populations declined progressively over 16 months of storage at 22 °C at both target a_w_ levels, but survival was consistently greater at lower a_w_ (Figure 1). At 0.42 ± 0.03 a_w_, the mean population decreased from 8.48 log CFU/g at month 0 to 2.70 log CFU/g at month 16 (Figure 1a), whereas at 0.26 ± 0.02 a_w_ the population decreased from 8.89 to 3.64 log CFU/g over the same period (Figure 1b). In both peanut butter treatments, a_w_ remained close to the intended target throughout storage after equilibration, varying around 0.42–0.45 in the higher-a_w_ treatment and around 0.25–0.27 in the lower-a_w_ treatment.

**Figure 2:**
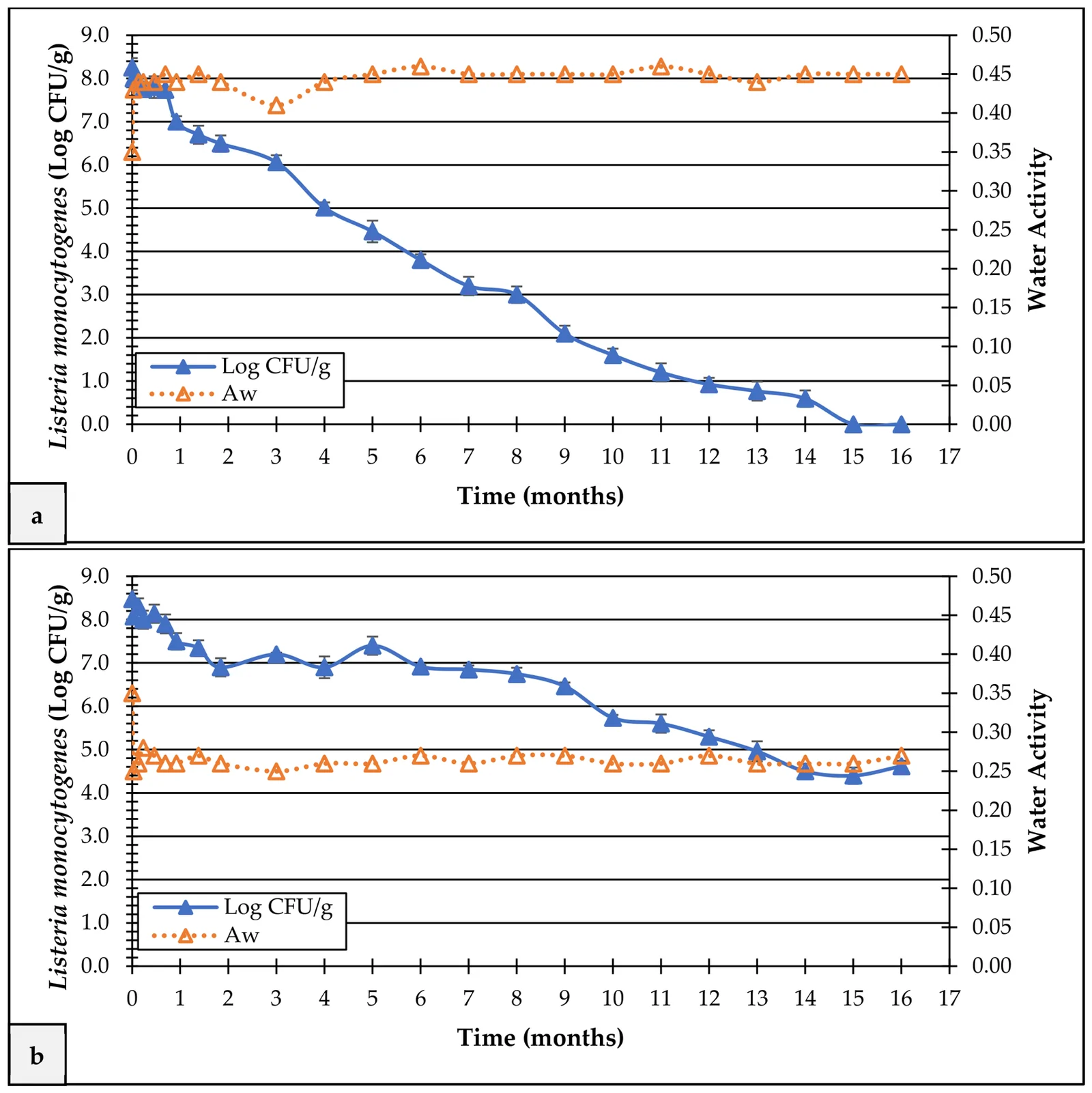
Survival of *Listeria monocytogenes* in whole egg powder at different water activity (a_w_) levels during 16 months of storage at 22 °C. (**a**) 0.44 ± 0.02 a_w_; (**b**) 0.27 ± 0.02 a_w_. The solid (dark blue) line shows the population of *L. monocytogenes*, while the dashed (orange) line represents the a_w_ of whole egg powder over storage time. Points represent mean log CFU/g values, and error bars represent the standard deviation of six replicates (n = 6; two biological replicates × three technical replicates per time point).

A similar protective effect of low a_w_ was observed in whole egg powder during storage at 22 °C (Figure 2). At 0.44 ± 0.02 a_w_, the mean *L. monocytogenes* population declined from 8.25 log CFU/g at month 0 to nondetectable levels by months 15–16 (Figure 2a), whereas at 0.27 ± 0.02 a_w_ the population decreased from 8.47 to 4.62 log CFU/g by month 16 (Figure 2b). Notably, although populations at 0.44 ± 0.02 a_w_ fell below the limit of detection by conventional plating, viability PCR (vPCR) analysis continued to detect viable cells, indicating the persistence of *L. monocytogenes* with intact membranes despite the absence of culturable cells. As in peanut butter powder, a_w_ remained relatively stable after equilibration, fluctuating around 0.44–0.46 in the higher-a_w_ treatment and 0.25–0.27 in the lower-a_w_ treatment. Collectively, these storage data show that reducing a_w_ increased long-term survival of *L. monocytogenes* in both matrices, with the effect being particularly pronounced in whole egg powder.

### 3.3. Model Discrimination Supported Nonlinear Thermal Inactivation Behavior

**Figure 3:**
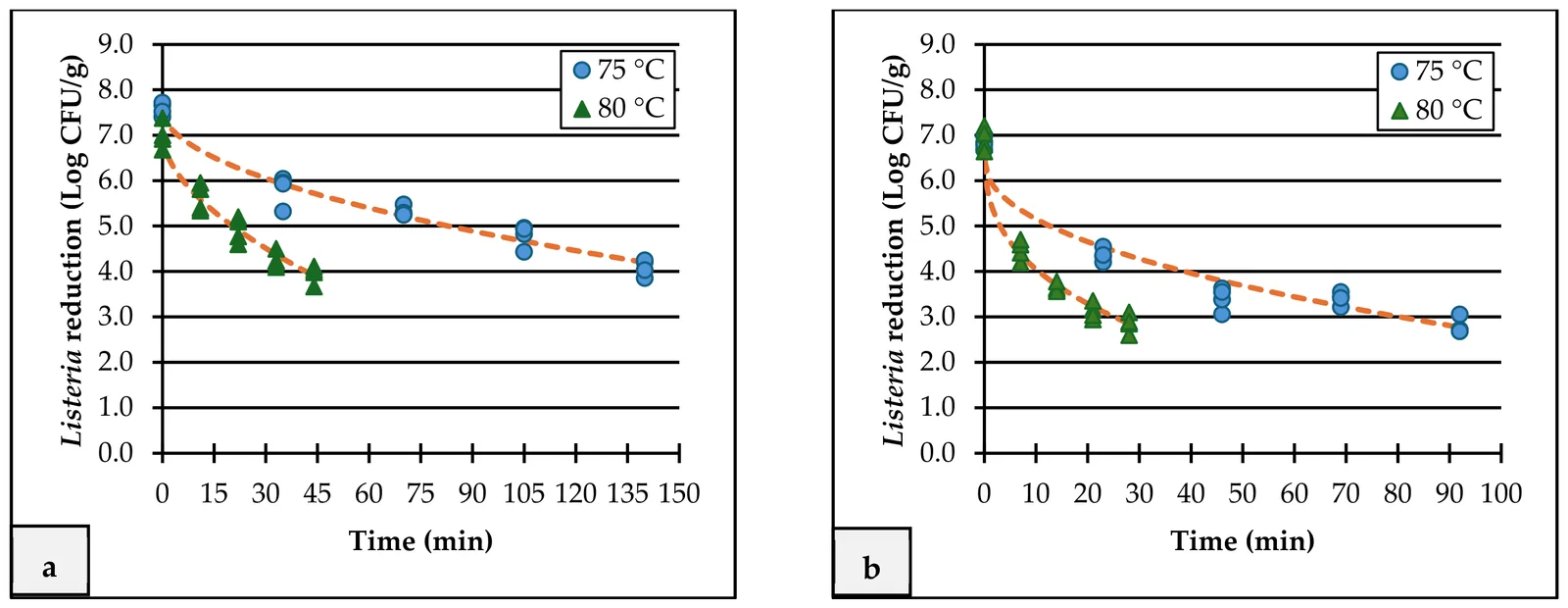
Thermal inactivation curves of *Listeria monocytogenes* in peanut butter powder equilibrated to (**a**) 0.25 a_w_ and (**b**) 0.45 a_w_, across selected temperatures. Points represent individual observations from two independent biological replicates, with three technical measurements per biological replicate at each treatment time (n = 6 observations per time point). The dashed (orange) lines represent fits of the selected Weibull model.

**Figure 4:**
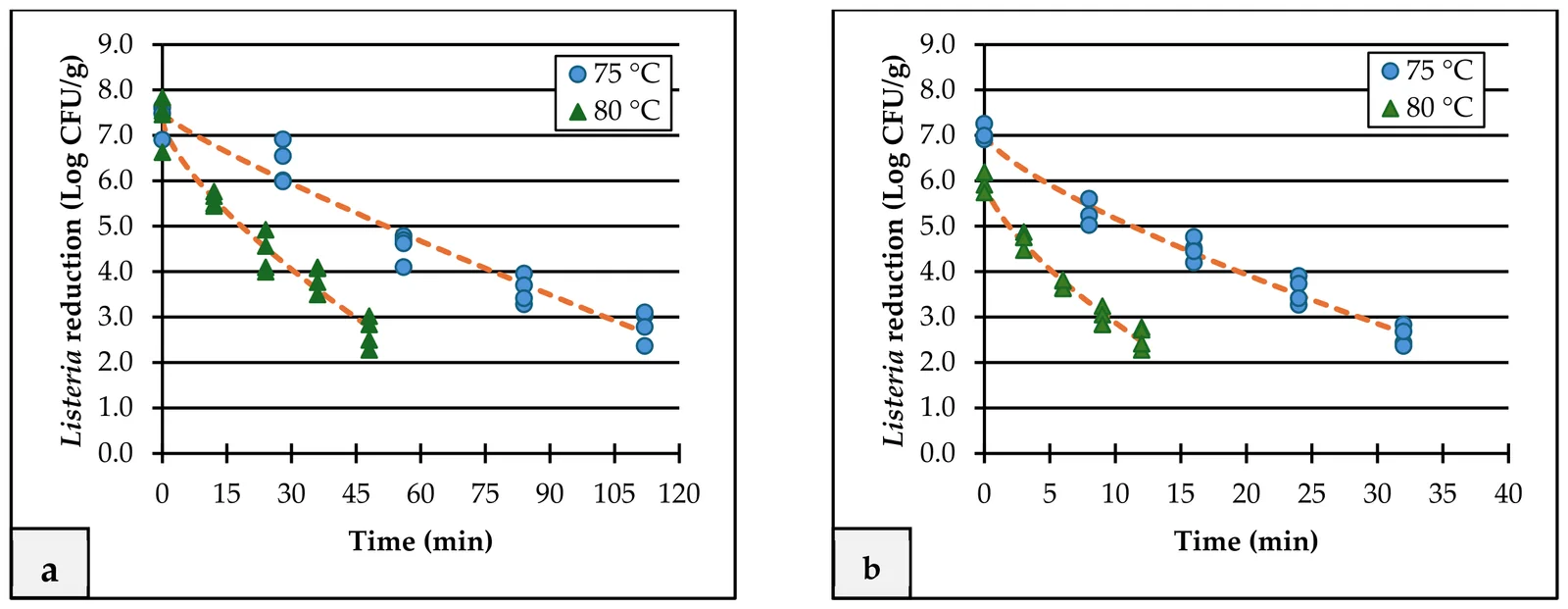
Thermal inactivation curves of *Listeria monocytogenes* in whole egg powder equilibrated to (**a**) 0.25 a_w_ and (**b**) 0.45 a_w_, across selected temperatures. Points represent individual observations from two independent biological replicates, with three technical measurements per biological replicate at each treatment time (n = 6 observations per time point). The dashed (orange) lines represent fits of the selected Weibull model.

Thermal inactivation curves for *L. monocytogenes* in both matrices showed clear departures from simple log-linearity, particularly under conditions where tailing was evident, and the selected Weibull fits visually tracked the observed decline patterns across all matrix–a_w_–temperature combinations (Figure 3 and Figure 4). In peanut butter powder, the Weibull model provided strong fits at all conditions (R^2^ = 0.939–0.977), although the biphasic model ranked first by AICc at 75 °C for both a_w_ levels, while the Weibull model ranked first at 80 °C for both a_w_ levels (Table 2 and Table 3). Importantly, for peanut butter powder at 75 °C/0.25 a_w_ and 75 °C/0.45 a_w_, the Weibull model remained competitive, with ΔAICc values of 0.668 and 1.262, respectively, indicating substantial support despite not being the top-ranked model by AICc.

Although AICc identified the biphasic model as the top-ranked model under several conditions, the Weibull model was retained as the common basis for cross-condition comparisons. This decision was not based on universal statistical superiority. Rather, the Weibull model had strong empirical support across all conditions, was within ΔAICc < 2 of the top-ranked model in the close comparisons, used fewer parameters than the biphasic model, and provided a common scale parameter for factorial analysis. In contrast, several biphasic fits produced boundary-adjacent fractions, large rate constants, and confidence intervals outside biologically admissible ranges, which limited parameter interpretability.

In whole egg powder, the Weibull model ranked first at 75 °C/0.25 a_w_, 80 °C/0.25 a_w_, and 75 °C/0.45 a_w_, and it ranked second only at 80 °C/0.45 a_w_, where the biphasic model had the lowest AICc (Table 4 and Table 5). Even in that case, the Weibull model remained strongly supported (ΔAICc = 0.380), and Weibull fits consistently showed high explanatory power across all treatments (R^2^ = 0.953–0.979). In contrast, the log-linear model generally showed inferior fit in both matrices, particularly in peanut butter powder at 0.45 a_w_, where R^2^ values were below 0.80, and AICc values were substantially poorer than those of the nonlinear models. Overall, these results indicate that the thermal inactivation behavior of *L. monocytogenes* in both low-moisture matrices was predominantly nonlinear, justifying the use of the Weibull model for subsequent D-value estimation and interpretation.

Several biphasic-model confidence intervals extended outside the admissible parameter ranges, including *f* < 0 or *f* > 1 and, in one condition, a negative lower confidence limit for an inactivation-rate constant. These intervals resulted from unconstrained asymptotic estimation and should not be interpreted as biologically feasible values. Rather, they indicated weak local identification of the biphasic parameters under the available sampling design. The point estimates and confidence intervals were retained to show model uncertainty, but mechanistic interpretation of the affected fractions and rate constants was avoided.

The biphasic fits also produced very small estimated fractions together with large rate constants under several conditions. This combination suggested parameter compensation: markedly different combinations of *f*, k_max1_, and k_max2_ could describe similar survivor-curve shapes. The resulting wide or boundary-crossing confidence intervals provided additional evidence of limited parameter identifiability and possible overparameterization relative to the number and distribution of observations. Consequently, the biphasic model was used primarily as a descriptive candidate model, and its individual parameters were not assigned direct biological meaning when uncertainty was large.

### 3.4. Weibull Parameter Estimates Reflected Greater Resistance at Lower a_w_ and Lower Temperature

The Weibull scale parameter (*δ*), which represents the time associated with a 1-log reduction under the fitted model, decreased as treatment temperature increased from 75 to 80 °C in both matrices, and it was generally higher at 0.25 a_w_ than at 0.45 a_w_ (Table 2 and Table 4). In peanut butter powder, *δ* decreased from 13.583 min at 75 °C/0.25 a_w_ to 6.062 min at 80 °C/0.25 a_w_, and from 1.211 min at 75 °C/0.45 a_w_ to 0.440 min at 80 °C/0.45 a_w_. In whole egg powder, δ decreased from 18.305 min at 75 °C/0.25 a_w_ to 5.308 min at 80 °C/0.25 a_w_, and from 4.375 min at 75 °C/0.45 a_w_ to 1.818 min at 80 °C/0.45 a_w_.

Across all Weibull fits, the shape parameter *β* remained below 1.0, ranging from 0.318 to 0.569 in peanut butter powder and from 0.673 to 0.868 in whole egg powder, indicating concave-downward survivor curves consistent with tailing behavior (Table 2 and Table 4). The lower *β* values observed in peanut butter powder, particularly at 0.45 a_w_, suggest more pronounced nonlinearity in this matrix. These parameter trends are consistent with the fitted curves shown in Figure 3 and Figure 4, and reinforce the conclusion that both a_w_ and matrix composition influenced the rate and pattern of thermal inactivation.

### 3.5. Weibull Scale Parameter (δ) Differed by Product, Temperature, and Water Activity

The Weibull scale parameter (*δ*; reported as geometric means with back-transformed 95% confidence intervals) showed a clear effect of treatment temperature within each a_w_ level and, in most cases, a significant effect of a_w_ within product–temperature combinations (Table 6). In peanut butter powder, the modeled time to first 1-log reduction at 0.25 a_w_ was significantly greater than those at 0.45 a_w_ at both 75 °C and 80 °C, as indicated by the row-wise uppercase letter comparisons (13.38 vs. 1.08 min at 75 °C; 5.82 vs. 0.44 min at 80 °C). Within the 0.25 a_w_ column, both products had larger *δ*-values at 75 °C than at 80 °C, whereas within the 0.45 a_w_ column, peanut butter powder had lower *δ*-values than whole egg powder at both temperatures.

Whole egg powder showed the same direction of a_w_ effect at 75 °C, with a significantly larger *δ*-value at 0.25 a_w_ than at 0.45 a_w_ (18.28 vs. 4.22 min), but this a_w_ effect was not significant at 80 °C (5.17 vs. 1.78 min; Table 6). Overall, the largest modeled time to the first 1-log reduction was observed for whole egg powder at 75 °C/0.25 a_w_ (18.28 min), followed closely by peanut butter powder at 75 °C/0.25 a_w_ (13.38 min), whereas the smallest *δ*-value was observed for peanut butter powder at 80 °C/0.45 a_w_ (0.44 min). These results demonstrate that lower a_w_ increased thermal resistance in both products, although the magnitude and statistical support of the a_w_ effect depended on both matrix and treatment temperature.

## 4. Discussion

The clearest biological signal in this study was the protective effect of lower water activity (a_w_) on *Listeria monocytogenes* survival. During storage at 22 °C, both matrices retained larger populations at the lower target a_w_, while during isothermal heating, Weibull scale parameter (*δ*) were consistently higher at 0.25 a_w_ than at 0.45 a_w_ for peanut butter powder and, at 75 °C, also for whole egg powder (Figure 1, Figure 2, Figure 3 and Figure 4; Table 6). This pattern agrees with previous studies showing greater pathogen persistence and thermal resistance at lower a_w_ in low-moisture foods, where reduced water availability limits cellular damage and mobility [5,6,23].

The 16-month storage data further demonstrated prolonged persistence at lower a_w_ in both matrices, particularly in whole egg powder, where culturable cells remained detectable at month 16 under the lower-a_w_ condition,, whereas counts declined to nondetectable levels under the higher-a_w_ condition (Figure 1 and Figure 2). Similar patterns of long-term persistence at reduced a_w_ have been reported in peanut-containing products, wheat flour, and almond meal [12,13,15]. Together with the thermal data, these results are consistent with the ability of desiccation-stressed *L. monocytogenes* to persist and tolerate subsequent lethal stresses [4].

Consistent with this interpretation, detection of *L. monocytogenes* by viability PCR (vPCR) in whole egg powder (a_w_ = 0.44 ± 0.02), despite direct plate counts below the detection limit, indicates the presence of membrane-intact cells that were not recovered by direct plating. However, PMA-based vPCR does not by itself demonstrate viability or a VBNC state. The discrepancy between vPCR and plating may reflect stressed or sublethally injured cells with intact membranes but reduced culturability. Similar observations have been reported in low-moisture foods [24,25]. Thus, direct plating, vPCR, and enrichment provide complementary information on culturability, membrane integrity, and recovery potential, respectively. From a food safety perspective, persistence of membrane-intact *L. monocytogenes* under low-a_w_ conditions warrants consideration, particularly if favorable conditions during subsequent handling or rehydration permit recovery [26,27].

While a_w_ was the dominant factor governing survival and thermal resistance, the data also showed that matrix composition shaped the magnitude of the response. At 0.45 a_w_, whole egg powder had larger *δ*-values than peanut butter powder at both 75 and 80 °C, whereas both matrices were more similar at 0.25 a_w_ (Table 6). Thus, the effect of a given a_w_ depended on the food microenvironment rather than a_w_ alone, consistent with previous reports of strong matrix dependence in low-moisture foods [6,17,28,29].

Despite the substantially greater lipid content in whole egg powder compared to peanut butter powder (Table 1), the protective effect of the matrix was not consistent across all conditions. This variability is supported by mechanistic evidence indicating that lipids’ role in *L. monocytogenes* thermal resistance is influenced by multiple factors, including temperature and matrix properties such as heat transfer characteristics, viscosity, structural organization, and cell localization within the food system [17]. Similar observations have been reported in nut butters and peanut-based products, where *L. monocytogenes* exhibits significantly greater heat tolerance in butters and spreads than in aqueous systems, underscoring the combined influence of low a_w_ and lipid-rich environments on enhancing microbial thermal resistance [12,30].

For the present study, the stronger protection observed in whole egg powder at 0.45 a_w_ may therefore reflect a combination of high fat, high protein, and matrix-specific microstructural effects rather than fat alone. In addition, animal-derived matrices may provide compatible solutes, particularly carnitine, that are important for osmotic adaptation in *L. monocytogenes* [31]. Carnitine and glycine betaine are preferred compatible solutes for this organism, and uptake systems such as OpuC are induced under osmotic stress, with regulation tied to the σ^B general stress response [10,11]. That mechanism offers a plausible explanation for why whole egg powder, an animal-derived matrix, showed relatively strong protection under some conditions despite having a higher native a_w_ than peanut butter powder before equilibration (Table 1).

Overall, the storage and thermal inactivation data support the concept that prior exposure to low-moisture conditions enhances resistance to subsequent lethal stresses. In this study, *L. monocytogenes* persisted for extended periods under low a_w_ during storage (Figure 1 and Figure 2), and these same conditions were associated with the highest *δ*-values during heat treatment (Table 6). This relationship is biologically plausible, as desiccation is known to activate global stress-response pathways in *L. monocytogenes*, including σ^B regulation, accumulation of compatible solutes, and cross-protection mechanisms that increase tolerance to osmotic, acid, oxidative, and thermal challenges [7,9]. Accordingly, the observed effect is unlikely to be explained solely by reduced heat transfer at low a_w_, but rather by the induction of a physiological state that is more resistant to heat-induced damage.

This mechanistic interpretation also helps explain why the magnitude of the a_w_ effect varied between matrices. The observed product × a_w_ interaction for *δ*-values suggests that the protective effect is shaped not only by the intensity of the desiccation stress but also by the compositional and structural features of the food system, such as the availability of compatible solutes and differences in lipid–protein organization. In other words, the cells were likely responding to the combined physicochemical environment rather than to a_w_ alone. This interpretation is consistent with the current literature describing *L. monocytogenes* persistence in low-moisture foods and related processing environments [6,7].

The model discrimination further supports this mechanistic interpretation. In both matrices, the log-linear model showed the poorest performance, whereas the Weibull and biphasic models consistently yielded better fits based on AICc, RMSE, and R^2^ (Table 2, Table 3, Table 4 and Table 5). That result is biologically plausible in low-moisture systems because desiccated populations are unlikely to be physiologically uniform. Instead, they likely consist of subpopulations with varying levels of stress tolerance, leading to the downward curvature and tailing observed in Figure 3 and Figure 4. The Weibull shape parameter values below 1.0 in both matrices are consistent with that interpretation and suggest that the surviving fraction became progressively more difficult to inactivate as treatment progressed [22,32]. From a process perspective, this is important because a log-linear assumption would tend to underestimate treatment time for higher log reductions when the survivor curves tail. The fact that the Weibull model remained first-ranked or strongly supported (ΔAICc < 2) across nearly all conditions indicates that it captured the response structure better than the classical first-order approach, especially under the dry, matrix-protective conditions where heat tolerance was greatest (Table 3 and Table 5). That conclusion aligns with several research studies on low-moisture food safety, showing that nonlinear primary models are often more appropriate when heat is applied to desiccation-stressed pathogens in complex dry matrices [21,29,33,34].

A key applied implication of these findings is that thermal inactivation behavior should not be extrapolated across low-moisture foods solely on the basis of temperature and nominal a_w_. The substantial differences in *δ*-values between the two matrices and the matrix- and temperature-dependent a_w_ effects (Table 6) support product-specific thermal validation that considers matrix composition and prior desiccation exposure. The persistence observed during storage also reinforces the importance of controlling contamination before and after processing. Collectively, these results support an integrated approach combining a_w_ control, sanitation, and matrix-specific thermal validation.

## 5. Conclusions

This study demonstrates that a_w_ is a primary determinant of both long-term survival and thermal resistance of *Listeria monocytogenes* in low-moisture foods, with lower a_w_ consistently promoting greater persistence during storage at 22 °C and larger modeled times to the first 1-log reduction (*δ*) during isothermal heating in both peanut butter powder and whole egg powder. Across both matrices, thermal resistance decreased as treatment temperature increased, but the magnitude of the a_w_ effect depended on the food matrix, indicating that matrix composition modified the protective effect of low a_w_ rather than acting independently of it.

The thermal inactivation data further showed that nonlinear behavior predominated, as the Weibull and biphasic models consistently outperformed the classical log-linear model across most treatment conditions, supporting the use of nonlinear modeling approaches for process evaluation in low-moisture systems. Overall, these findings show that *L. monocytogenes* can remain highly stable in dry food matrices and can exhibit substantial heat resistance under low-a_w_ conditions, underscoring the need for matrix-specific and a_w_-specific lethality validations when designing thermal control strategies for low-moisture foods.

## Figures and Tables

**Table 1:** The proximate analysis composition of selected low-moisture foods.

Composition	Peanut Butter Powder ^a^	Whole Egg Powder ^a^
Water activity (a_w_)	0.14 ± 0.01	0.21 ± 0.01
Moisture (%)	2.75 ± 0.19	3.95 ± 0.15
Crude Protein (%)	39.83 ± 0.02	44.95 ± 0.04
Crude Fat (%)	9.74 ± 0.10	36.86 ± 0.17
Crude Fiber (%)	2.01 ± 0.09	0.19 ± 0.02
Carbohydrates (%)	41.37 ± 0.14	9.51 ± 0.18
Ash (%)	6.31 ± 0.01	4.73 ± 0.01

^a^ Mean ± SD, n = 3. a_w_ measured at 22 °C.

**Table 2:** Parameter estimates (±95% CI) and selected goodness-of-fit metrics for primary inactivation models describing the inactivation of *Listeria monocytogenes* in peanut butter powder during isothermal treatments.

Log-Linear Model								
Parameters ^a^	75 °C/0.25 a_w_		80 °C/0.25 a_w_		75 °C/0.45 a_w_		80 °C/0.45 a_w_	
	Value	95% CI ^b^	Value	95% CI ^b^	Value	95% CI ^b^	Value	95% CI ^b^
k_max_ (min^−1^)	0.023	(0.019, 0.026)	0.067	(0.056, 0.078)	0.039	(0.030, 0.048)	0.132	(0.101, 0.163)
R^2^/AICc	0.900/−34.249		0.884/−34.135		0.789/−12.187		0.793/−11.366	
**Biphasic model**								
*f*	0.929	(0.874, 0.984)	0.084	(−0.075, 0.242)	0.002	(−0.000, 0.004)	0.002	(−0.001, 0.006)
k_max1_ (min^−1^)	4.772	(4.772, 4.772)	0.106	(0.056, 0.156)	0.030	(0.014, 0.045)	0.115	(0.046, 0.183)
k_max2_ (min^−1^)	0.038	(0.031, 0.044)	0.386	(0.077, 0.695)	0.260	(0.222, 0.299)	0.868	(0.685, 1.052)
R^2^/AICc	0.974/−55.073		0.938/−40.536		0.981/−54.812		0.967/−42.400	
**Weibull model**								
δ	13.583	(6.435, 20.731)	6.062	(2.296, 9.829)	1.211	(−0.044, 2.466)	0.440	(−0.067, 0.947)
β	0.520	(0.407, 0.633)	0.569	(0.400, 0.738)	0.318	(0.241, 0.395)	0.338	(0.243, 0.433)
R^2^/AICc	0.968/−54.405		0.939/−44.092		0.977/−53.55		0.967/−45.159	

^a^ $a_{w}$—water activity; $k_{max}$—inactivation rate (min^−1^); f—fraction of labile bacteria; $k_{max1}$—inactivation rate constant of the labile fraction (min^−1^); $k_{max2}$—inactivation rate constant of the stable fraction (min^−1^); δ—scale parameter; β—shape parameter; $R^{2}$—coefficient of determination; AICc—corrected Akaike information criterion. ^b^ Model intervals were not boundary-constrained; therefore, some limits extend outside the admissible parameter space (e.g., *f* < 0, *f* > 1, or *δ* < 0). These limits indicate weak parameter precision and should not be interpreted as biologically feasible values.

**Table 3:** Model discrimination for *Listeria monocytogenes* inactivation in peanut butter powder during isothermal treatments.

Model	Model Performance/Goodness of Fit ^a^						
	RMSE	R^2^	R^2^_Adj_	BIC	AICc	ΔAICc	Rank
**Treatment: 75 °C**/**0.25 a_w_**							
Biphasic	0.193	0.974	0.967	−53.757	−55.073	0.000	1
Weibull	0.213	0.968	0.962	−52.918	−54.405	0.668	2
Log-linear	0.378	0.900	0.888	−32.963	−34.249	20.824	3
**Treatment: 80 °C**/**0.25 a_w_**							
Biphasic	0.278	0.938	0.921	−39.220	−40.536	3.556	2
Weibull	0.275	0.939	0.927	−42.605	−44.092	0.000	1
Log-linear	0.379	0.884	0.870	−32.850	−34.135	9.957	3
**Treatment: 75 °C**/**0.45 a_w_**							
Biphasic	0.195	0.981	0.976	−53.496	−54.812	0.000	1
Weibull	0.217	0.977	0.972	−52.063	−53.550	1.262	2
Log-linear	0.656	0.789	0.764	−10.901	−12.187	42.625	3
**Treatment: 80 °C**/**0.45 a_w_**							
Biphasic	0.265	0.967	0.959	−41.084	−42.400	2.759	2
Weibull	0.268	0.967	0.961	−43.672	−45.159	0.000	1
Log-linear	0.669	0.793	0.769	−10.081	−11.366	33.792	3

^a^ RMSE—Root mean square error; $R^{2}$—coefficient of determination; $R_{adj}^{2}$—adjusted coefficient of determination; BIC—Bayesian information criterion; AICc—corrected Akaike information criterion; ΔAICc—delta-corrected Akaike information criterion.

**Table 4:** Parameter estimates (±95% CI) and selected goodness-of-fit metrics for primary inactivation models describing the inactivation of *Listeria monocytogenes* in whole egg powder during isothermal treatments.

Log-Linear Model								
Parameters ^a^	75 °C/0.25 a_w_		80 °C/0.25 a_w_		75 °C/0.45 a_w_		80 °C/0.45 a_w_	
	Value	95% CI ^b^	Value	95% CI ^b^	Value	95% CI ^b^	Value	95% CI ^b^
k_max_ (min^−1^)	0.043	(0.038, 0.047)	0.093	(0.081, 0.105)	0.134	(0.121, 0.146)	0.288	(0.257, 0.319)
R^2^/AICc	0.949/−31.792		0.932/−29.331		0.962/−43.429		0.948/−45.546	
**Biphasic model**								
*f*	0.999	(0.988, 1.010)	0.922	(0.740, 1.103)	0.813	(0.649, 0.976)	0.020	(−0.029, 0.070)
k_max1_ (min^−1^)	0.116	(0.087, 0.145)	0.425	(0.099, 0.751)	4.436	(4.436, 4.436)	0.341	(0.117, 0.565)
k_max2_ (min^−1^)	0.035	(−0.082, 0.151)	0.168	(0.112, 0.224)	0.266	(0.235, 0.297)	1.006	(0.766, 1.246)
R^2^/AICc	0.962/−32.722		0.957/−32.677		0.980/−50.153		0.983/−61.301	
**Weibull model**								
δ	18.305	(9.873, 26.737)	5.308	(2.391, 8.224)	4.375	(2.871, 5.878)	1.818	(1.228, 2.408)
β	0.868	(0.660, 1.076)	0.691	(0.526, 0.855)	0.741	(0.620, 0.862)	0.673	(0.564, 0.781)
R^2^/AICc	0.953/−32.852		0.956/−35.186		0.978/−51.659		0.979/−60.922	

^a^ $a_{w}$—water activity; $k_{max}$—inactivation rate (min^−1^); f—fraction of labile bacteria; $k_{max1}$—inactivation rate constant of the labile fraction (min^−1^); $k_{max2}$—inactivation rate constant of the stable fraction (min^−1^); δ—scale parameter; β—shape parameter; $R^{2}$—coefficient of determination; AICc—corrected Akaike information criterion. ^b^ Model intervals were not boundary-constrained; therefore, some limits extend outside the admissible parameter space (e.g., *f* < 0, *f* > 1, or *δ* < 0). These limits indicate weak parameter precision and should not be interpreted as biologically feasible values.

**Table 5:** Model discrimination for *Listeria monocytogenes* inactivation in whole egg powder during isothermal treatments.

Model	Model Performance—Goodness of Fit ^a^						
	RMSE	R^2^	R^2^_Adj_	BIC	AICc	ΔAICc	Rank
**Treatment: 75 °C**/**0.25 a_w_**							
Biphasic	0.338	0.962	0.952	−31.405	−32.722	0.130	2
Weibull	0.374	0.953	0.945	−30.304	−32.852	0.000	1
Log-linear	0.391	0.949	0.943	−31.566	−31.792	1.060	3
**Treatment: 80 °C**/**0.25 a_w_**							
Biphasic	0.338	0.957	0.946	−31.361	−32.677	2.509	2
Weibull	0.344	0.956	0.947	−33.699	−35.186	0.000	1
Log-linear	0.427	0.932	0.924	−28.046	−29.331	5.855	3
**Treatment: 75 °C**/**0.45 a_w_**							
Biphasic	0.219	0.980	0.975	−48.836	−50.153	1.507	2
Weibull	0.228	0.978	0.974	−50.172	−51.659	0.000	1
Log-linear	0.300	0.962	0.958	−42.144	−43.429	8.230	3
**Treatment: 80 °C**/**0.45 a_w_**							
Biphasic	0.165	0.983	0.978	−59.985	−61.301	0.000	1
Weibull	0.181	0.979	0.975	−59.434	−60.922	0.380	2
Log-linear	0.285	0.948	0.942	−44.260	−45.546	15.756	3

^a^ RMSE—Root mean square error; $R^{2}$—coefficient of determination; $R_{adj}^{2}$—adjusted coefficient of determination; BIC—Bayesian information criterion; AICc—corrected Akaike information criterion; ΔAICc—delta-corrected Akaike information criterion.

**Table 6:** Weibull-modeled time to first 1-log reduction (*δ*) for *Listeria monocytogenes* in peanut butter powder and whole egg powder at different water activity levels and temperatures.

Product	Treatment Temperature	0.25 a_w_ *		0.45 a_w_ *	
		*δ* (min)	95% CI	*δ* (min)	95% CI
Peanut Butter	75 °C	13.38 ^aA^	(9.18, 19.52)	1.08 ^bB^	(0.29, 3.98)
Powder	80 °C	5.82 ^bA^	(2.63, 12.89)	0.44 ^bB^	(0.17, 1.13)
Whole Egg	75 °C	18.28 ^aA^	(16.34, 20.45)	4.22 ^aB^	(2.54, 6.99)
Powder	80 °C	5.17 ^bA^	(2.15, 12.42)	1.78 ^aA^	(1.07, 2.96)

* a_w_—Water activity; CI—Confidence interval. Values are Weibull scale parameter (*δ*; min) expressed as geometric means with back-transformed 95% confidence intervals, calculated from two independent biological-replicate curves per treatment (n = 2). Lowercase letters compare means within each a_w_ column, and uppercase letters compare a_w_ levels within the same row. Means sharing the same letter size are not significantly different (*p* > 0.05).

## Data Availability

The original contributions presented in this study are included in the article. Further inquiries can be directed to the corresponding author.
